# TMSmap – Software for Quantitative Analysis of TMS Mapping Results

**DOI:** 10.3389/fnhum.2018.00239

**Published:** 2018-07-09

**Authors:** Pavel A. Novikov, Maria A. Nazarova, Vadim V. Nikulin

**Affiliations:** ^1^Centre for Cognition and Decision Making, Institute for Cognitive Neuroscience, National Research University Higher School of Economics, Moscow, Russia; ^2^Department of Neurology, Max Planck Institute for Human Cognitive and Brain Sciences, Leipzig, Germany; ^3^Neurophysics Group, Department of Neurology, Campus Benjamin Franklin, Charité—Universitätsmedizin Berlin, Berlin, Germany

**Keywords:** motor cortex, transcranial magnetic stimulation (TMS), cortical mapping, earth mover's distance (EMD), overlap, interpolation on the sphere, free software

## Abstract

The use of the MRI-navigation system ensures accurate targeting of TMS. This, in turn, results in TMS motor mapping becoming a routinely used procedure in neuroscience and neurosurgery. However, currently, there is no standardized methodology for assessment of TMS motor-mapping results. Therefore, we developed TMSmap—free standalone graphical interface software for the quantitative analysis of the TMS motor mapping results (http://tmsmap.ru/). In addition to the estimation of standard parameters (such as the size of cortical muscle representation and the center of gravity location), it allows estimation of the volume of cortical representations, excitability profile of the cortical surface map, and the overlap between cortical representations. The input data for the software includes the coordinates of the coil position (or electric field maximum) and the corresponding response in each stimulation point. TMSmap has been developed for versatile assessment and comparison of TMS maps relating to different experimental interventions including, but not limited to longitudinal, pharmacological and clinical studies (e.g., stroke recovery). To illustrate the use of TMSmap we provide examples of the actual TMS motor-mapping analysis of two healthy subjects and one chronic stroke patient.

## Introduction

Transcranial magnetic stimulation (TMS) is a non-invasive approach for studying brain organization in humans. In TMS time-varying magnetic fields generate electrical currents in the targeted brain regions resulting in the activation of the neuronal tissue (Ruohonen and Karhu, 2010). The magnetic field strongly attenuates with increasing distance from the coil and, therefore, the brain areas stimulated with TMS are located rather superficially at a distance of about 2–4 cm under the skin (Groppa et al., 2012), which corresponds roughly to the gray-white matter border. In order to identify the stimulated cortical area, a precise location of the coil should be determined with respect to the individual's brain anatomy. This can be achieved with a combination of magnetic resonance imaging (MRI) data, and devices tracking the position of the subject's head and TMS coil, so-called navigated TMS (nTMS) approach which provides a navigation with millimeter accuracy (Krieg, 2017). Such spatial accuracy is particularly useful for TMS cortical mapping where the brain is stimulated at small and usually regularly spaced points. Theoretically, any response to TMS elicited from a specific cortical point, can be used for TMS mapping. In principle, TMS mapping can be specified with the following two parameters: (1) type of TMS protocol: for instance, single pulse, or paired pulse TMS—routinely used for motor TMS mapping, repetitive TMS - used for speech areas and so on; (2) type of the measured biological activity: electromyography (EMG) for motor cortex mapping, behavioral responses (phosphenes, reaction times, hit-rate, errors, etc.), electroencephalography (EEG), functional MRI (fMRI) etc. Such a variety of protocols and neurophysiological measures indicates that TMS mapping represents a promising approach for the non-invasive investigation of the brain in different fields of neuroscience.

Presently, however, only presurgical motor and speech nTMS mapping has received widespread use (Krieg et al., 2017). Yet the use of TMS mapping for the investigation of cortical neuroplastic changes, after surgery, during rehabilitation, training, or for any other longitudinal investigations, is still limited both in clinical and basic research environments. This is despite the fact that TMS compared, for example, to functional MRI, reflects the link between the structure and function in a direct causal way and usually requires less compliance from a subject. There are several reasons for such situation. Firstly, even for TMS motor mapping, there is no general agreement about the exact parameters describing cortical representations. Moreover, even most commonly used parameters such as areas, volumes, the location of centers of gravity (CoGs), and hotspots have not been validated for being reliable measures in test-retest studies (Kraus and Gharabaghi, 2016). Eventually, even for the extensively studied field of stroke recovery the lack of the standardized methodology leads to considerable difficulties when summarizing the findings of the publications with TMS motor mapping (Lüdemann-Podubecká and Nowak, 2016). Secondly, despite the long history of TMS motor mapping, one should still develop a standardized toolbox or software for the quantitative analysis of TMS mapping results. Yet, a unified workflow would alleviate results' comparisons across sessions, subjects and studies.

In order to address these challenges, we present TMSmap—free standalone graphical interface software for quantitative analysis of the TMS mapping results (http://tmsmap.ru/, registration number RU 2016614899, 11.05.2016, a freeware license). It provides an integrative approach for the complex assessment and visualization of TMS motor mapping results. The software includes assessment of the standard features of TMS maps, such as areas and volumes of the cortical representations, CoGs, and hotspots, as well as novel parameters such as cortical representations' excitability profiles and the overlaps between the cortical representations—showing, for example, coactivation of the different muscles.

The purpose of this article is to describe the software options and workflow, primarily, for the quantification of TMS motor mapping data. To illustrate the programs' capabilities, we use examples of nTMS multi-muscle motor mapping in two healthy subjects and in one chronic stroke patient with hand motor deficit.

## Method—software

**Figure 1:**
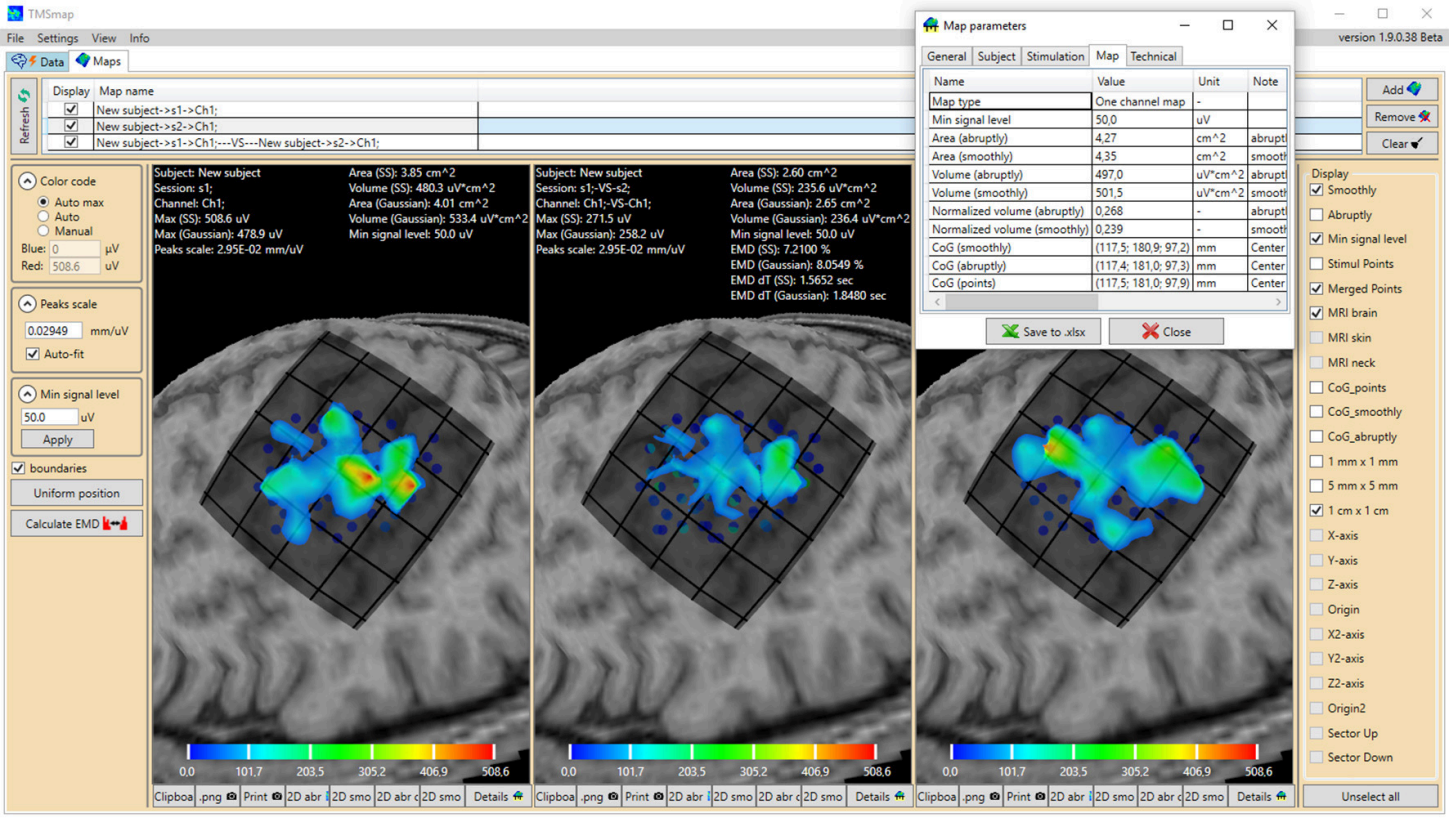
General view of the software interface. Upper panel represents two main tabs of the program: “Data” (preprocessing) and “Maps” (3D map construction and comparison). The tab “Maps” is chosen, cortical representation maps for two muscles (left and right maps) and their overlap (in the middle) are represented. Left panel contains the parameters of the map construction and has a button for EMD calculation. Right panel represents the features which can be vizualized. For each map the low panel represents the possibility to generate pictures and tables of the results. A table with the main information about the map is shown for one of the maps. Color bars are representing the amplitudes of the MEPs in microvolts.

TMSmap software is standalone software with a graphical interface (Figure 1). It was written using WPF (C# and XAML), based on .NET Framework 4.5.2, and it supports Microsoft Windows operating system version 7 or higher. TMSmap is a freeware: one can download and use it free of charge. In this section, we present TMSmap possibilities and general data workflow. We will also briefly describe methods and approaches for data analysis and visualization. As this article is not intended to be a user manual, we invite the reader to visit the website http://tmsmap.ru/ for further detailed information including video demonstrations.

**Figure 2:**
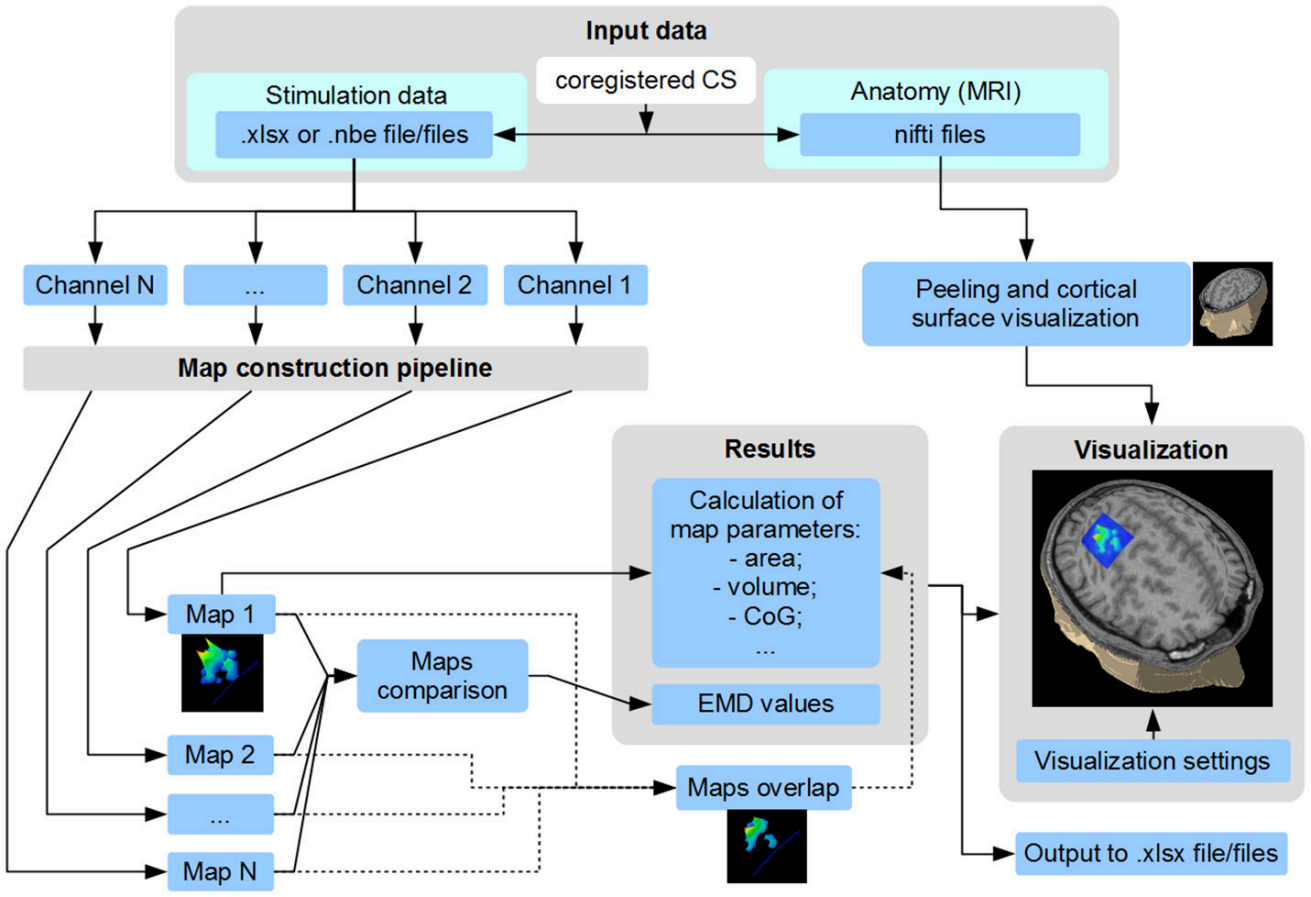
Scheme of TMSmap possibilities. CS, coordinate system; CoG, center of gravity; EMD, earth mover's distance.

TMSmap allows a fast construction and visualization of the 3D maps of cortical representations and a calculation of the parameters that are most commonly used in TMS mapping analysis such as: areas of representation, centers of gravity (CoG) locations, hotspots locations, and volumes of representation. In addition, we introduce several new mapping approaches: (1) a quantitative comparison of the excitability profiles for several cortical representations (located closely, or at a distance) by a metric called Earth Mover's Distance (EMD); (2) analysis of the overlaps between any two maps (leading to a creation of a new map). General scheme of the TMSmap possibilities, including an assessment of the standard and novel parameters of TMS motor maps, is presented in Figure 2.

### Input data

The input data for the software includes structural MRI and the actual results of the stimulation. MRI data is not absolutely necessary for the map reconstruction; it is needed primarily for the visualization of the relationships between the constructed map and the cortical structure. MRI data should be presented in the .nifti format (.img, .hdr files). Both structural MRI data and the stimulation coordinates may be either in the individual coordinates or in the template brain coordinate system, for example, in the MNI coordinate system, in cases of using preprocessed normalized data as in the study of Niskanen et al. (2010).

Data should contain the coordinates of the stimulation points which, in the simplest case, it can be just coil's coordinates on the scalp, as well as the coordinates of the estimated electric field (EF) maximum in the brain. Such coordinates can be obtained using different methodologies: using the line-of-sight approach (like in Raffin et al., 2015), or using EF maximum coordinates precalculated in the neuronavigation system like in Krieg et al. (2017), or using an offline approach with a sophisticated realistic volume conductor modeling of the head [e.g., utilizing a software presented in http://simnibs.de/ like in Bungert et al. (2017)]. For each stimulation point there are associated responses from any number of the recording channels. Current version of TMSmap is primarily designed for motor mapping data. In this case the channels should contain the information about the MEP characteristics such as peak-to-peak amplitude, latency, duration, number of peaks etc. However, other types of the parameters, for example “Yes”, “No” e.g., when inducing phosphenes or error types—can also be used as a response value. Stimulation data can be presented either in .xlsx file or in a text file of .nbe format in case of using Nexstim navigation system. It should be mentioned that TMSmap can be used for the purposes of viewing and editing of the stimulation data files (including .nbe files).

A detailed step-by-step procedure of the map construction is provided in the tutorials available at the website.

### TMSmap functions

#### Brain anatomy visualization

The definition of the skin surface is based on the data about the tissue density across voxels. The peeling is performed at the minimum distance sufficient for the visualization of all the stimulation points. Then the cortical landscape is visualized based on the tissue density differences between the cortex and the cerebro-spinal fluid.

#### Construction and visualization of the map surface

**Figure 3:**
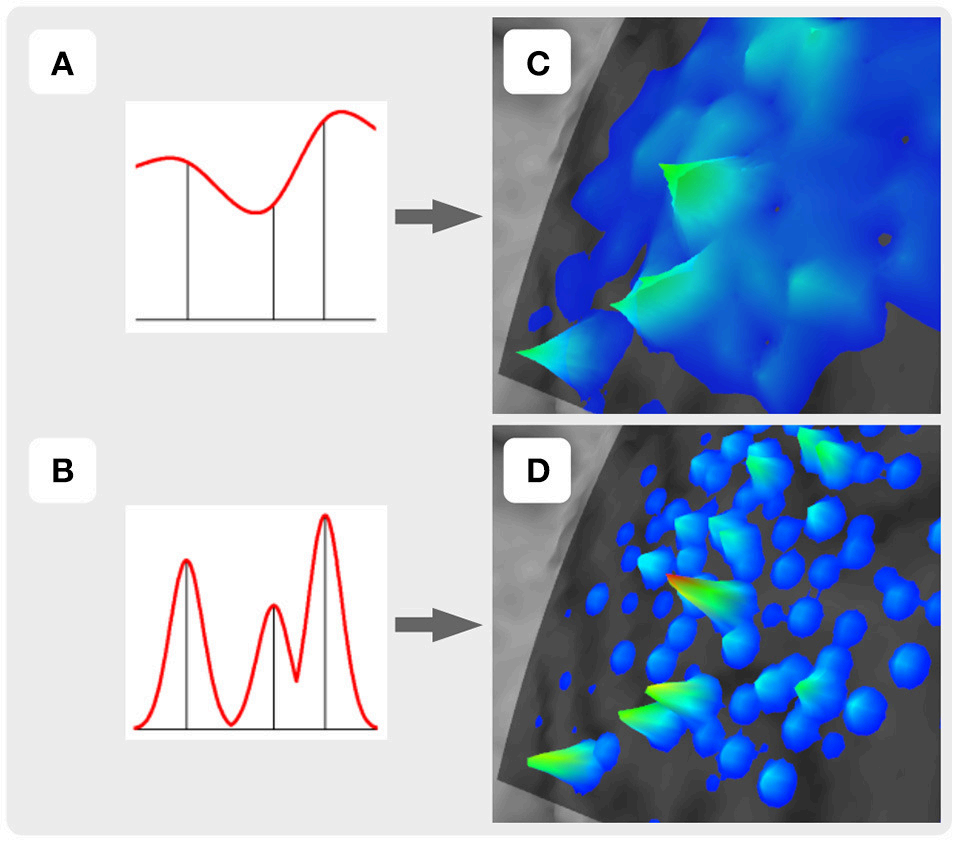
Two approaches of the map construction used in TMSmap: smoothly **(A)** and abruptly **(B)** changing response functions. **(C)** and **(D)** are specific examples for **(A)** and **(B)**, respectively.

For the construction of the representation surface, TMSmap uses two different approaches. In the first it is assumed that the response function is changing relatively smoothly among the stimulation points (Figure 3A). In this case for the surface construction TMSmap uses an approach allowing to create a maximally smooth surface going through all the points—approximation based on smoothing (ABOS) method (Dressler, 2009). Another approach for the map surface reconstruction, realized in the TMSmap, is based on the assumption that the response function may change rather abruptly from one point to another. In this case, the constructed surface would be built as a combination of the kernel surfaces defined by the exponential function created from each stimulation point; this approach would be further referred to as a Kernel approach (Figure 3B). Based on the physical principles of TMS it is not possible to stimulate one point without the stimulation of the adjacent ones (Ilmoniemi and Kicić, 2010). Thus, the approach based on the smoothly changing function is preferable for TMS mapping data analysis—this being in agreement with the data presented in the literature (Julkunen, 2014). A Kernel approach is still included in the software since we anticipate in the future a usage of high-resolution TMS with the next generation of the stimulators providing more focal stimulation (Koponen et al., 2017). In addition, TMSmap may be also utilized for motor mapping during invasive stimulation of the cortex (for neurosurgical purposes) where the spatial resolution of the stimulation is much higher.

**Figure 4:**
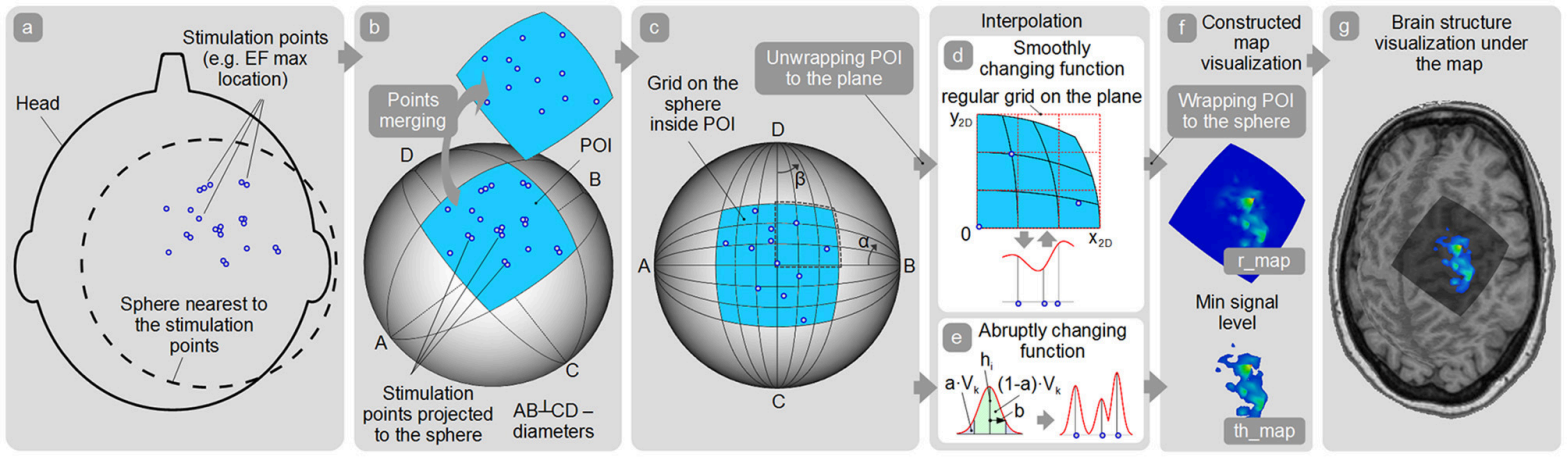
Map construction pipeline. **(a)** Finding the sphere nearest to the stimulation points; **(b)** Patch of interest (POI) creation around the projected stimulation points and points spatial merging; **(c)** Quasi-regular grid creation inside the POI; **(d,e)** Interpolation among the points in case of smoothly and abruptly changing function approaches; **(f)** r_map and th_map visualization; **(g)** Visualization of the representation on the cortex.

A general schema of the map surface construction consists of the following steps:

1. Obtain coordinates of all the stimulation points with the corresponding response values (e.g., MEP peak-to-peak amplitude). Optionally, a maximum of the induced EF (if available) can be taken into the account for weighting the response values.
2. All the stimulation points should be used for fitting the closest spherical surface which is found using the least squares method (Figure 4a). All stimulation points are projected to this sphere (Figure 4b). For maps' comparison all the stimulation points should be fitted to the same spherical surface. Consequently, this part of the sphere surface is defined as shown in Figure 4b for the further analysis. This part of the sphere surface would be further referred to as a patch of interest (POI).
3. Spatial filtering (merging) is applied to the points (optionally). Therefore, new merged points with the averaged locations and averaged/maximal response values are created (Figure 4b). Using this option it is possible to utilize only merged points with sufficient number of the repetitions for map construction.
4. Quasi-regular grid inside the POI is constructed (Figure 4c).
5. In case of abruptly changing function assumption (Figure 4e) Kernel approach is used for map construction. A height of the surface in any arbitrary point inside the POI is defined according to the formula:
  $$\text{h​}\left({\text{A}}_{\alpha ,\beta }\right)={\text{max}}_{i=1...\text{N}}\left({\text{h}}_{\text{i}}\cdot a^{\left(\frac{{\text{r}}^{\text{2}}\left({\text{A}}_{\alpha ,\beta },{\text{A}}_{\text{i}}\right)}{{\text{b}}^{\text{2}}}\right)}\right),$$
  where
  A_α,β_ – point with the angle coordinates (α, β);
  N – a number of the merged projected stimulation points;
  h_i_ – averaged response value in the merged point A_i_;
  a – the portion of the whole kernel volume located outside the radius b. Hereby, b is the navigation accuracy in (1 − a) portion of all the cases. For example, the default TMSmap settings for a - 0.05 and for b - 2 mm. It means that only 5% of the whole kernel volume would be more than 2mm further from the stimulation point, indicating 95% probability for the real stimulation point to be at this given area;
  r(A_α,β_, A_i_) – geodesic distance between points A_α,β_ and A_i_;
6. In case of using a smoothly changing function approach the found POI with the merged projected points is unwrapped to the plane (Figure 4d). Arbitrary POI point A_α,β_ will have plane coordinates:
  $${\text{x}}_{\text{2D}}\left(\alpha ,\beta \right)=\text{k}\cdot \cos\theta ,{\text{y}}_{\text{2D}}=\text{k}\cdot \sin\theta ,$$
  where
  $$\begin{array}{l} \text{k}=\text{R}\cdot \arccos\left(\frac{\cos\alpha \cdot \cos\beta }{\sqrt{1-\sin^{2}\alpha \cdot \sin^{2}\beta }}\right);\\ \theta =\{\begin{array}{c} \arctan\left(\frac{\tan\alpha }{\tan\beta }\right)+\pi \cdot \text{H}(-\beta ),\beta \neq 0\\ \text{sign}(\alpha )\cdot \pi /2,\beta =0 \end{array}; \end{array}$$
  R – Radius of the sphere where the stimulation points are projected;
  H – Heaviside step function.
  A regular grid is created around the unwrapped POI on the plane. Grid's element size is not a user-customized parameter. Additionally, user can define a maximal radius of the stimulation point influence which is a distance where an approximated map surface is reaching zero level (default TMSmap settings: 5 mm).
  At this stage map construction on the plane is performed using ABOS-approach (Dressler, 2009). After that a regular grid with the POI is wrapped back to the sphere:
  $$\alpha =\text{arctan }(\frac{{\text{x}}_{3\text{D}}}{{\text{z}}_{3\text{D}}}\;),\;\beta =\text{arctan}(\frac{{\text{y}}_{3\text{D}}}{{\text{z}}_{3\text{D}}}),$$
  where
  $$\begin{array}{l} {\text{x}}_{3\text{D}}=\frac{\text{R}\cdot \text{sin}\frac{\text{s}}{\text{R}}}{\sqrt{1+{\left(\frac{{\text{y}}_{2\text{D}}}{{\text{x}}_{2\text{D}}}\right)}^{2}}},{\text{y}}_{3\text{D}}={\text{x}}_{3\text{D}}\cdot \frac{{\text{y}}_{2\text{D}}}{{\text{x}}_{2\text{D}}},{\text{z}}_{3\text{D}}=\text{R}\cdot \text{cos}\frac{\text{s}}{\text{R}},\\ \text{s}=\sqrt{{{\text{x}}_{2\text{D}}}^{2}+{{\text{y}}_{2\text{D}}}^{2}}. \end{array}$$
7. The constructed map is defined as a raw map – r_map for the further analysis (Figure 4f).
8. An additional color coding of the created response map is possible using a continuous color scale from blue (min response value level) to red (max response value level).
9. A minimum signal level surface is created based on the user-defined threshold (e.g., MEP amplitude equal to 50 μV for standard area calculation or a percentage of the largest MEP within the map for highest excitability regions identification etc.). This surface is concentric to the sphere where stimulation points were projected. Only the part of the response surface which is above this minimum signal level surface will be taken for the further analysis, it will be defined as a thresholded map—th_map (Figure 4f).
10. Optionally, brain structure is visualized under the constructed map (Figure 4g).

The default values of the map construction parameters proposed in TMSmap are explained in the manual. In order to compare results of the different studies, all the parameters should be ideally the same. Of course, this does not prevent the user from exploring different parameters' values.

#### Construction of the maps' overlap

It is possible to choose any of the two constructed maps (e.g., channel 1 and channel 2) to create their overlap map (Figure 2). The overlap of the other maps can be calculated iteratively for any two pairs of the maps. The overlap maps have the same set of the properties as the channel's maps.

#### Area and volume calculation

Map area is defined as an area of the projection of the th_map to POI. Map volume is calculated as a volume under this th_map surface.

#### CoG calculation

CoGs locations for raw data and for the constructed 3D-maps are calculated according to the following equations:

$$\begin{array}{l} {\text{ x}}_{\text{CoG}}^{\text{raw}}=\frac{\sum_{i=1}^{\text{N}}\left({\text{h}}_{\text{i}}\;\cdot {\text{ x}}_{\text{i}}\right)}{\sum_{\text{i}=1}^{\text{N}}{\text{h}}_{\text{i}}};\\ {\text{x}}_{\text{CoG}}^{\text{th}\_\text{map}}=\frac{\sum_{i=1}^{{\text{N}}_{\text{E}}}\left({\text{h}}_{\text{E}\_\text{i}}\;\cdot {\text{ s}}_{\text{i}}\;\cdot {\text{ x}}_{\text{E}\_\text{i}}\right)}{\sum_{i=1}^{{\text{N}}_{\text{E}}}\left({\text{h}}_{\text{E}\_\text{i}}\;\cdot {\text{ s}}_{\text{i}}\right)}, \end{array}$$

where in case of raw data

N – number of the merged points;

h_i_ and x_i_ – averaged response value and x coordinate of the merged point, respectively;

and in case of th_map

N_E_ – number of the grid's elements belonging to the th_map;

h_E_i_, s_i_, x_E_i_ – height of the constructed map above the grid's element, area and x coordinate of the center of the grid's element, consequently.

#### Creation of the color-coded 2D maps

**Figure 5:**
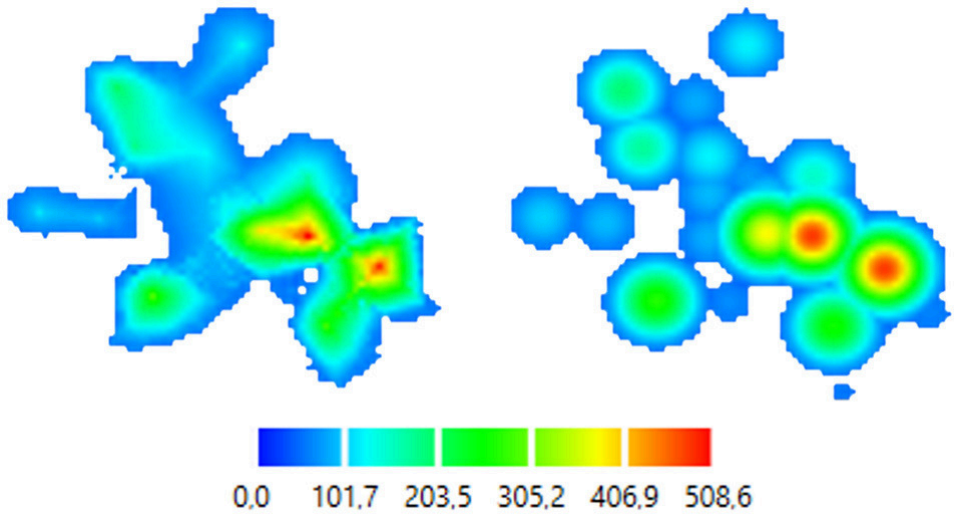
Color-coded 2D map representations. **Left**—smoothly changing function approach (ABOS based map construction), **Right**—the same data, abruptly changing function approach (Kernel based map construction). Color bar is representing the amplitudes of the MEPs in microvolts.

There is an option to create separate files of color-coded pictures for each map (Figure 5). These files are saved in .png format for the further analysis.

#### Maps 3D profiles comparison—EMD

Map surface could be characterized by its individual 3D profile. TMSmap allows numerical comparison of the profiles of both th_map and the distributions of the raw (not interpolated) response values using the so-called Wasserstein metric or EMD (Rubner et al., 1998, 2000). EMD is a minimum amount of work needed to shift one distribution to another. The notion of “work” in case of maps comparison would be a geodesic distance between the stimulation points. EMD may be useful to numerically differentiate maps located nearby to each other. These maps might be similar in terms of the standard parameters such as areas and volumes but still varying due to the complexity of their excitability profiles. Implementation of the EMD metric in TMSmap is based on the modification of the open-source C++ code (http://ai.stanford.edu/~rubner/emd/default.htm#LOG). In TMSmap the two maps should be normalized by volume before calculating EMD between them. Then EMD is calculated and represented as a relative value – a percentage of the EMD between two extreme maps each consisting of the only one peak maximally separated from each other in the limits of the individual size of the “active” area. For each subject individually we define “active area” in the following manner: we include any stimulated point where at least one muscle in at least one session had a response with the amplitude higher than a chosen threshold (e.g., MEP amplitude > 50 μV). A maximum distance between the points in such active area is taken as an individual constant for the EMD normalization.

#### Results output

All calculated parameters are represented in the results tables (Figure 1) and can be copied or saved as an .xlsx file.

#### Visualization

TMSmap allows offline visualization of the multiple features of the TMS mapping results such as:

1. Stimulation points;
2. POI;
3. Stimulation points projected to the POI;
4. Spatially merged points with color-coded averaged response values;
5. Minimum signal level surface – a user-customized threshold surface;
6. r_map and th_map built based on one of the two possible approaches (ABOS or Kernel);
7. 3 types of CoGs based on the above-mentioned approaches;
8. Overlaps of any of the two maps;
9. 2D color-coded figures of any map, including overlap maps;
10. Different size grids on the POI.

All mapping features can be visualized in relation to an individual brain MRI. All visualization parameters can be represented independently or in parallel.

## Examples

In this section, we will provide examples of TMS motor mapping using datasets from two healthy subjects and one chronic ischemic stroke patient with a favorable hand motor recovery. We previously presented an example of the earlier version of the software for TMS motor mapping in healthy and stroke subjects in the PhD thesis of one of the authors (Nazarova, 2015). All TMS investigations were carried out in accordance with the safety TMS guidelines (Rossi et al., 2009), subjects participating in the TMS mapping procedure gave a written informed consent in accordance with the Declaration of Helsinki. All subjects were screened for contraindications to TMS (Rossi et al., 2009) before the consenting process. Experiments were approved by the local Ethics Committees of the Research Center of Neurology and Higher School of Economics (Moscow) and TMS motor mapping was performed in the Research Center of Neurology, Moscow. A Nexstim eXimia stimulator with nTMS-compatible electromyography (EMG) device, navigation software, and a figure-of-eight coil (Focal Bipulse, Nexstim Plc, Helsinki, Finland) was used for the stimulation. Structural T1 MRIs required for the navigation were acquired with 1.5 T MR-scanner Siemens Magnetom Avanto (T1 weighted; 1 mm thickness; sagittal orientation; acquisition matrix 256 × 256). As a first step a “hotspot” of the cortical representation of the abductor pollicis brevis (APB) muscle was found in the primary motor cortex. The resting motor threshold (RMT) for the given “hotspot” was determined as a minimal stimulator output producing contralateral APB MEPs with a minimal amplitude being 50 μV in a resting muscle, in 5 out of 10 given stimuli (Rossini et al., 1994). The intensity of the stimulation during the mapping procedure was always kept at 110% of the RMT for APB. Minimal time lag between the stimuli was 3 s. Further details for the mapping procedure are described below.

We used data from two healthy right-handed male volunteers (30 and 28 years old). For these volunteers two TMS motor mapping sessions separated by 7 days were performed (Day 1 and Day 2). The sessions consisted of nTMS mapping of the cortical representation of the three right hand muscles: APB, abductor digiti minimi (ADM) and extensor digitorum communis (EDC). Each of the two mapping sessions included 5 sub-sessions each including 55 or 53 TMS pulses for subject 1 and 2, respectively. The stimulation nodes were pre-set using a virtual MRI-based grid, so that each of the point was stimulated in a pseudo-random order five times. Day 2 points were stimulated in the exactly same order as on the Day 1. An error of the navigation for each cortical spot was kept below 2 mm. RMT for APB was 35 and 31% for the volunteer 1 and 2, respectively.

For the third example we used the data from a chronic stroke patient (male, 55 years old, 20 months after the incident) with the cortical-subcortical ischemic lesion in the left hemisphere and favorable upper limb motor recovery (55 by Fugl-Meyer upper extremity scale). The RMTs for APB was 51% for the unaffected hemisphere (UH) and 75% for the affected hemisphere (AH). A total amount of the points was 120 for the UH and 125 for the AH, each spot was stimulated only once.

The MEP amplitudes were calculated online in the eXimia software. During the initial preprocessing EMG data were visually inspected. Only a few MEPs had to be rejected due to concurrent muscle contraction. We used precalculated EF maximum location coordinates provided by the navigation software. Further analysis was performed in the TMSmap. The following possibilities of the TMSmap are shown: a visualization of the excitability profiles of the TMS muscle cortical representations and their overlaps; a possibility to visualize the stimulation points and the merged points based on the chosen criteria for spatial filtering; calculation of the areas and volumes of the motor maps and their overlaps; comparison of the 3D excitability profiles of the different sessions and different muscles using EMD metric.

**Figure 6:**
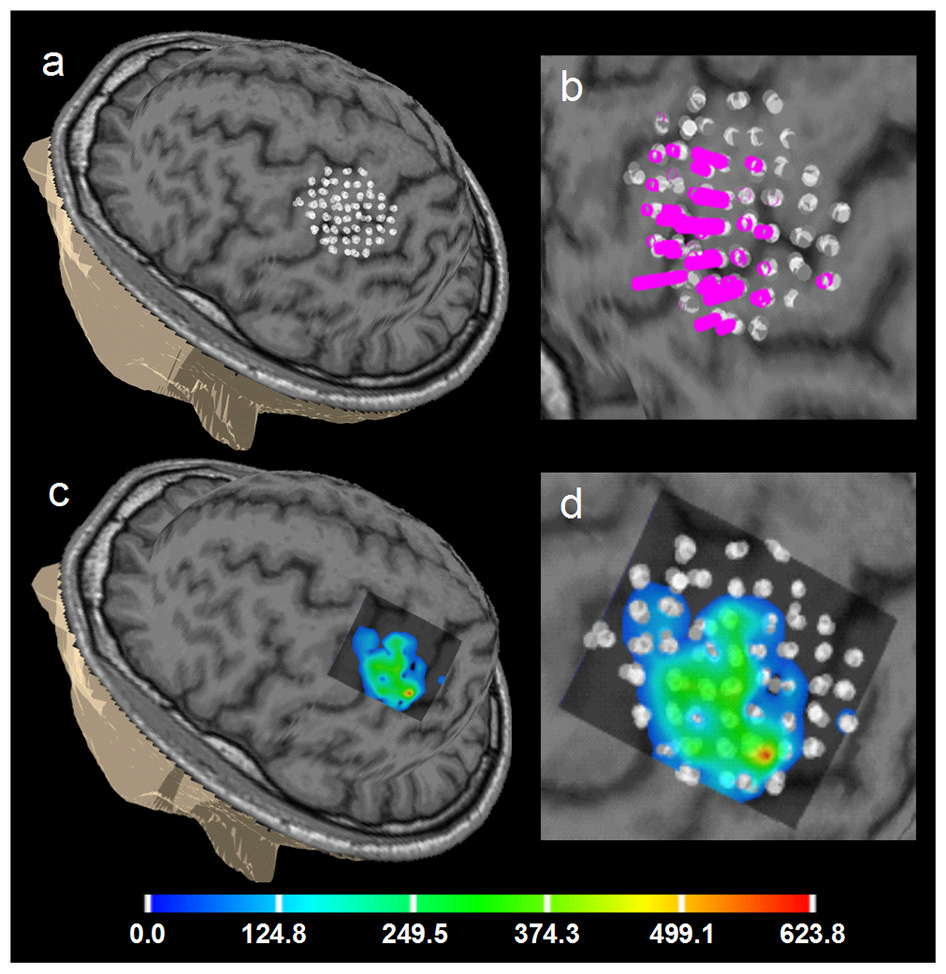
Healthy volunteer 1. ABOS based visualization of the APB cortical representation, a summation of 5 sessions of the first day, not merged stimulation points are visualized to show the accuracy of the stimulation of the same point in 5 sub-sessions. **(a)** points visualized on the brain surface; **(b)** its zoomed view with the perpendiculars from the points, height of the perpendiculars reflects the MEPs amplitudes. **(c)** ABOS based APB map reconstruction on the brain surface and **(d)** its zoomed view. Color scale representing amplitudes of the MEPs in microvolts is shown.

**Figure 7:**
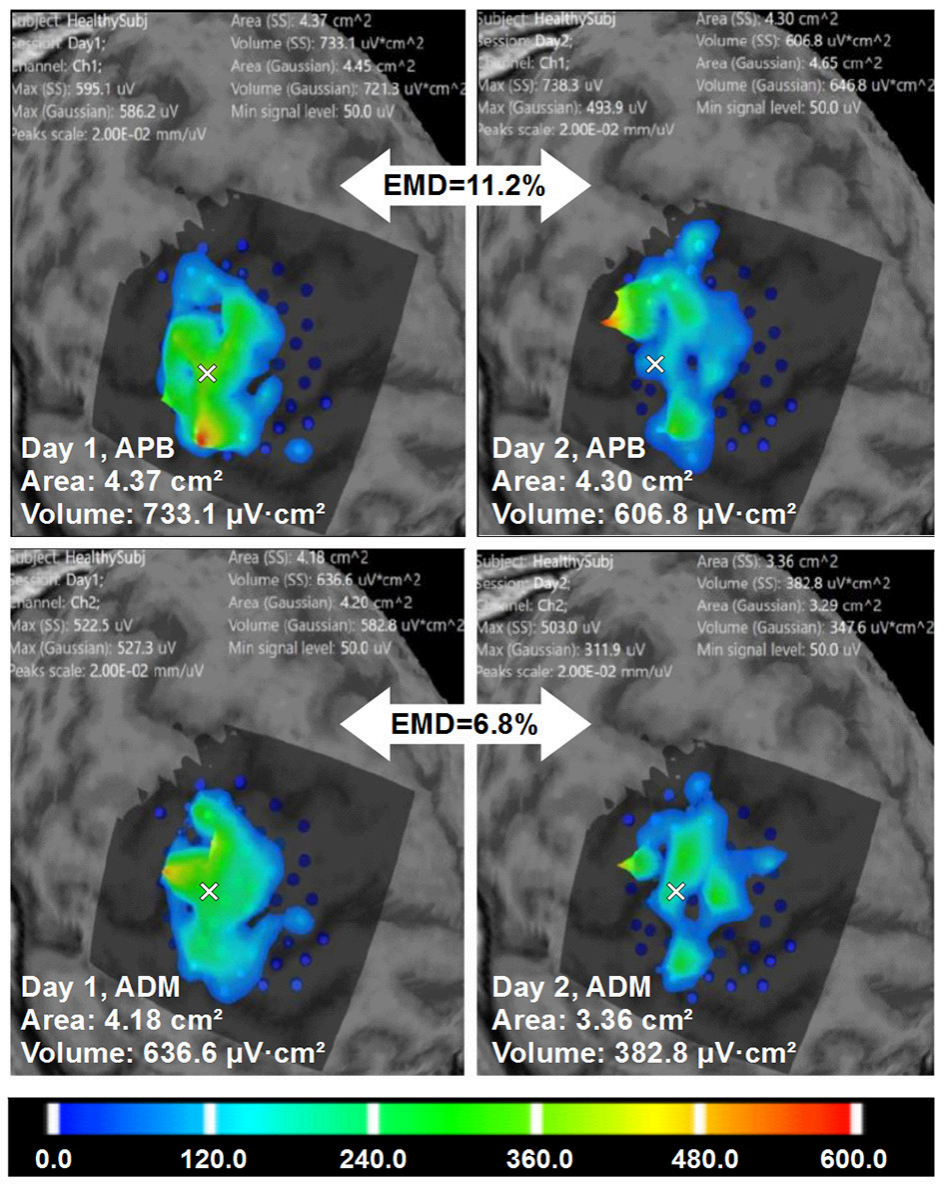
Healthy volunteer 2. The visualization of the ABOS based surface reconstruction of APB and ADM maps in both days (already based on five sessions for each day, merged points with more than two repetitions in each). CoGs are shown with a white cross. It is worth noting that APB maps in Day 1 and Day 2 are more similar in terms of areas and volumes. However, the excitability profiles reliability is higher for ADM cortical representation. Color scale reflecting amplitudes of the MEPs in microvolts is shown.

Figure 6 shows an example of the right APB cortical surface map for the first healthy subject. Stimulation points without applying spatial filtering (merging) are shown to demonstrate an accuracy of the repeated stimulation of the same point in 5 sub-sessions. In Figure 7 an example of the test-retest mapping data from the healthy volunteer 2 is presented. Here spatial filtering (merging) of the stimulation points was used. The similarity of the 3D profiles of the different maps measured using EMD metric is shown.

**Figure 8:**
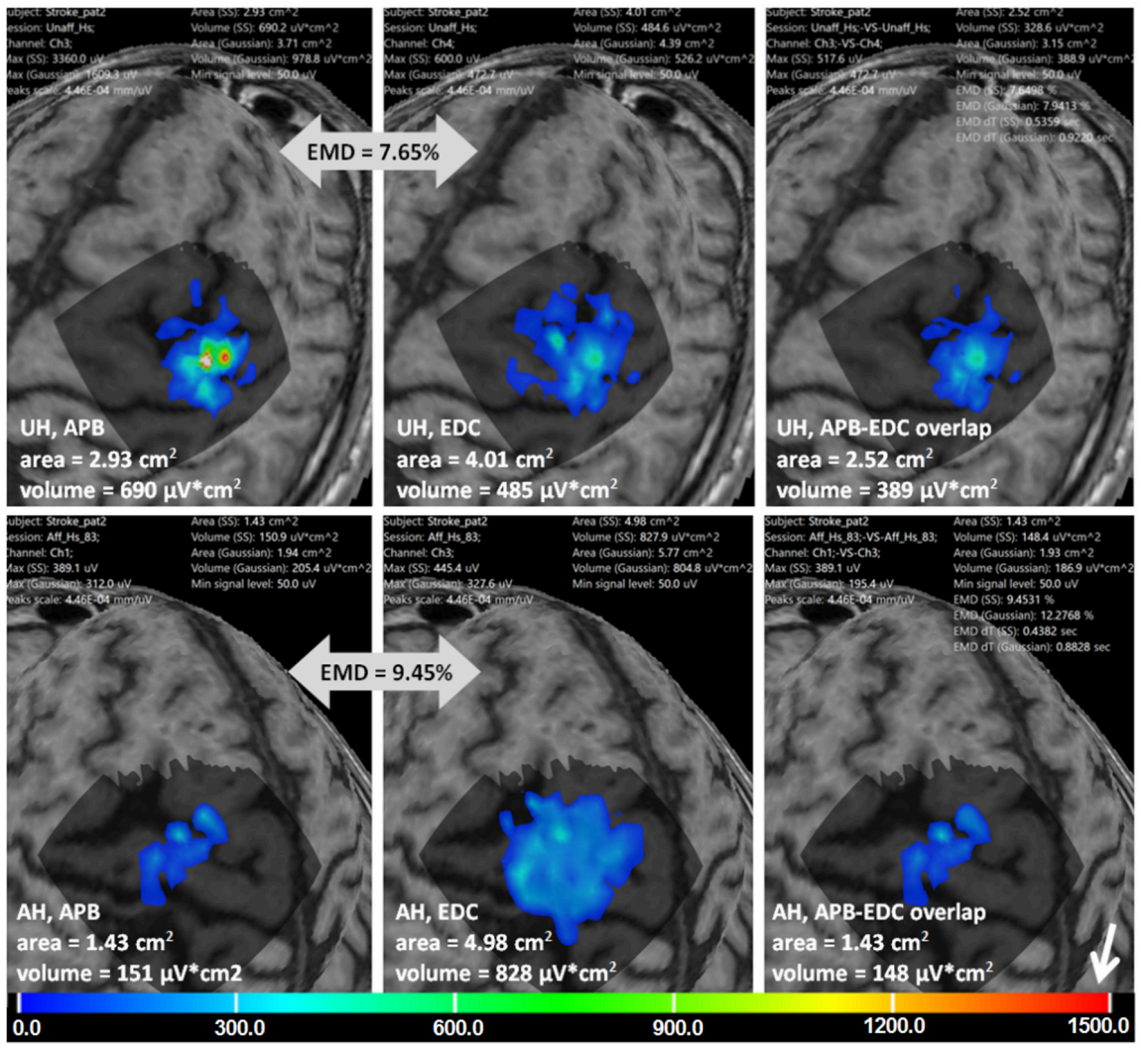
Stroke patient. APB and EDC cortical representations and their overlaps using ABOS based surface reconstruction are created for both hemispheres. Maps' areas and volumes are visualized. Relative EMD values among APB and EDC representations are shown (big gray arrows). Color scale representing amplitudes of the MEPs in microvolts is shown with a small white arrow.

Figure 8 is an example of multi-muscle TMS mapping in the ischemic stroke patient. A greater difference between APB and EDC representations in the AH compared to the UH could be observed, it is manifested as a shrinkage of the APB and an extension of the EDC cortical representation. EMD values reflecting the differences in the topographies are also shown.

## Discussion

We have developed and introduced TMSmap—novel standalone software with a graphical interface for the quantitative analysis of the nTMS mapping results. To the best of our knowledge until now, there was no such versatile software for the analysis of TMS mapping results. Previous approaches were primarily based on custom-made scripts for Matlab (Niskanen et al., 2010; Kraus and Gharabaghi, 2016; van de Ruit and Grey, 2016) which makes the comparison and generalization of the results between groups a challenge. In this article we described the functionality of TMSmap and methods used for the construction and quantitative assessment of the cortical surface maps. Consequently, we illustrated its performance for the analysis of TMS motor maps in two healthy subjects and in one stroke patient. TMSmap was used for the assessment of similarities of cortical representations of different muscles and of same muscles in different days.

Here we focused primarily on the motor nTMS mapping as one of the most common applications of nTMS mapping (Ruohonen and Karhu, 2010; Lefaucheur and Picht, 2016). It is important to mention that until now, even for the estimation of a muscle's cortical representation area there is no standard approach (Julkunen, 2014) and its reliability in test-retest studies is rather questionable (Kraus and Gharabaghi, 2016). Therefore, the use of a standardized tool covering diverse steps of TMS mapping pipeline would facilitate comparison among studies, which is needed for multi-center studies and meta-analysis relating to TMS motor mapping in different conditions.

Moreover, despite being the most widely used metric, TMS cortical representation of a muscle is a rather challenging biological concept, especially when considering well-known principles of the motor cortex organization such as divergence and convergence (Schieber, 2001; Capaday et al., 2013; Nazarova and Blagovechtchenski, 2015). Apparently, measuring just an area of a given muscle cortical representation doesn't take into account these principles. TMS mapping results should not be interpreted as if the neuronal activation occurs at only one small brain point but rather that the induced motor responses are obtained for given coordinates of the coil or for the coordinates of the strongest values of the induced EF. TMS mapping still might be used for more comprehensive studies of motor cortex organization. This could be achieved for example by assessing relationships among the cortical representation of different muscles and by analyzing the regions where TMS produces simultaneous responses in several muscles. Such an approach takes into account both divergence and convergence phenomena. Indeed, in animal studies it was shown that around 50% of the cortico-motoneuronal cells recorded with microelectrodes facilitate at least one proximal and at least one distal muscle (McKiernan et al., 1998). Moreover, it was demonstrated that depending on the limb position, microstimulation at the same point may evoke activity in different muscles (Graziano, 2006). Interestingly, a similar effect was demonstrated during one spot TMS, where a concept of “selectivity ratio” was introduced, defined as the amplitude of the MEP elicited from the muscle depending on whether it acts as an agonist or antagonist in the following movement (Gerachshenko et al., 2008; Uehara et al., 2015). However, there are still only a few studies on TMS mapping dedicated specifically to the muscle representation overlap phenomenon. It is clear that such overlap is a prominent phenomenon (Wassermann et al., 1993; Devanne et al., 2006). Moreover, it was shown that there was, in fact, a difference in the extent of such overlap in dominant and non-dominant hemispheres (Melgari et al., 2008). In addition, it was demonstrated that the extent of this overlap can be changed in some pathological conditions like dystonia or chronic pain (Schabrun et al., 2009, 2015). One of the prominent hypotheses states that the amount of the overlap among different populations of cortical motor cells may represent a neural substrate for creating muscle synergies (Capaday et al., 2013). This, in turn, may be extrapolated to the TMS representations' overlaps. Moreover, there is already some evidence for this idea (Schabrun et al., 2009, 2015; Massé-Alarie et al., 2017). Thus, the continuation of the TMS investigation of cortical representations' overlaps appears to be a promising approach. Our software includes calculations of all parameters of the overlaps among different cortical representations, thus, allowing an estimation of corresponding plastic changes in longitudinal studies.

Another important parameter proposed in TMSmap is based on a convergence principle of motor cortex organization. It relates to the excitability profile of muscle cortical representation. The convergence principle indicates that different loci along the pre-central gyrus contain separate representations of the same muscle (Schieber, 2001). In animal studies it was clearly shown that the pyramidal neurons relating to a single digit muscle are widely distributed in the motor cortex and may even be found in the regions which are traditionally known to contain shoulder representation (Rathelot and Strick, 2006). In many TMS studies it was shown that the representation of a single muscle is widely distributed over the motor cortex (Wassermann et al., 1992; Lotze et al., 2003; Melgari et al., 2008). It was reported that muscle representation can have discrete islands of relatively stable responses (Littmann et al., 2013) and parts of the representation with higher excitability so-called “peaks of excitability” may be physiologically relevant for the understanding of motor outputs (Massé-Alarie et al., 2017). However, still in many TMS studies the variability among MEPs amplitudes in the cortical representation is rather ignored (Sollmann et al., 2013; Ruit et al., 2014). Recently it has been reported that in the case of TMS motor mapping consisting of only seven cortical spots along the central sulcus, it was possible to trace an excitability profile of the responses from several hand muscles characterized by partial somatotopy both during rest and during the isometric contraction of one of the muscles (Raffin et al., 2015). However, such line mapping may not be sufficient for mapping purposes in clinical studies considering the fact that even hotspots of the hand muscles can be located not on the “hand knob” (Ahdab et al., 2016). Therefore, a distributed excitability profile analysis including a wide grid of the stimulated points might be more advantageous. The question of the biological meaning of any parameter is tightly connected with its stability in normal conditions. In TMS map we proposed the way to analyze similarity of such excitability profiles using EMD, a metric which allows evaluation of the dissimilarity between two multi-dimensional distributions (Rubner et al., 1998, 2000; Haufe et al., 2008). Thus, the next step would be to investigate “the phenomenon of the excitability profile reliability” in a test–retest study—exemplary results are presented here for one of the healthy subjects.

TMSmap may be widely used for purposes of quantitative analysis and offline visualization of TMS motor mapping results, including longitudinal studies. Standardizing the procedure of map construction and their parameters would lead to an easier comparison of results, thus, being particularly relevant for studying reorganization in the course of a disease, rehabilitation or training. Indeed, until now in order to investigate TMS motor maps, the changes were analyzed either by visual assessment (Mäkelä et al., 2013) or using parameters such as areas of the representations, CoGs location and a few other characteristics alternating from study to study (Littmann et al., 2013; Ruit et al., 2014; Sankarasubramanian et al., 2015; Kraus and Gharabaghi, 2016). Multi-parametric assessment of the TMS motor maps including standard and novel parameters accessible in TMSmap may reveal more subtle changes in cortical organization. The simplest example of TMSmap usage is a classical TMS motor mapping with computation of MEPs amplitudes elicited by single pulse TMS applied to different cortical points. At the same time, any other parameter, detected with TMS-EMG approach, such as the latency or duration of MEP, the extent of paired-pulse TMS phenomena, intensity necessary to induce a silent period etc. may also be easily utilized for motor map reconstruction in TMSmap. Such comprehensive analysis of motor maps would be especially valuable in fields of neurorehabilitation and neuroenhancement where even small modifications of the map might be important for estimating the effects of intervention. Indeed, TMS mapping has already been shown to have a potential diagnostic/therapeutic value in pathological conditions such as stroke (Lüdemann-Podubecká and Nowak, 2016), amyotrophic lateral sclerosis (Chervyakov et al., 2015), dystonia (Thickbroom et al., 2003; Quartarone, 2013), multiple sclerosis (Thickbroom et al., 2005), pain therapy (Nurmikko et al., 2016) etc, as well as in healthy subjects under experimental conditions such as immobilization, training or neuromodulation (Ngomo et al., 2012; Boudreau et al., 2013) or in special populations such as musicians (Elbert and Rockstroh, 2004), sportsmen (Hänggi et al., 2015) or surgery trainees. TMSmap may supposedly increase this potential. Finally, TMSmap capabilities are not restricted to the purposes of TMS motor mapping. Considering the fact that the necessary input data includes anatomical coordinates and any type of numerical/rank response, the software can be used for 3D map construction of other responses relating to behavioral performance, EEG, fMRI etc.

## Limitations and outlook

A cortical surface map construction is at the core of TMSmap, thus leading to the standardization of the mapping results. Evidently, other factors such as accuracy of the navigation system, as well as stimulation procedure itself are important sources of the TMS results' variability. However, just excluding the variability relating to map construction and parameters' calculation, should be beneficial for the assessment of TMS results. One limitation of TMSmap is that currently it has been tested with only three available TMS navigation systems: two commercial ones: Nexstim, Localite and one open-source—InVesalius neuronavigation (https://github.com/invesalius/invesalius3/tree/master/navigation/mtc_files). The latter two provide only information about the coil position in contrast to the Nexstim system, where in addition one has precalculated EF maximum coordinates based on the spherical model (Krieg, 2017). Further updates to the program will include compatibility with other navigation systems and the implementation of the existing types of EF distribution modeling (e.g., like Pitkänen et al., 2017). Currently, EF maximum coordinates (calculated with another software presented in http://simnibs.de/) can be used. In the future, we plan to provide ranges for the expected normal fluctuation of standard and novel parameters of the motor TMS maps in healthy subjects utilizing present and future reproducibility studies.

Link to the website http://tmsmap.ru/ – software for quantitative analysis of TMS mapping results.

## Author contributions

PN developed the software, developed some of the methods used in the software, tested the software, wrote the manuscript, and agreed to be accountable for all aspects of the work. MN generated the idea, supervised the whole project, performed TMS mapping experiments, tested the software, wrote the manuscript and agreed to be accountable for all aspects of the work. VVN participated in the development of the software, discussion and writing of the manuscript, he agreed to be accountable for all aspects of the work. PN and MN share the first authorship and have contributed equally to the study.

### Conflict of interest statement

The authors declare that the research was conducted in the absence of any commercial or financial relationships that could be construed as a potential conflict of interest.

## Abbreviations

ABOS: approximation based on smoothing
ADM: abductor digiti minimi
AH: affected hemisphere
APB: abductor pollicis brevis
CS: coordinate system
CoG: center of gravity
EDC: extensor digitorum communis
EEG: electroencephalography
EF: electric field
EMD: earth mover's distance
EMG: electromyography
fMRI: functional magnetic resonance imaging
MEP: motor evoked potential
MRI: magnetic resonance imaging
nTMS: navigated transcranial magnetic stimulation
POI: patch of interest
r_map: raw map
RMT: resting motor threshold
TMS: transcranial magnetic stimulation
th_map: thresholded map
UH: unaffected hemisphere.
